# Insulin Resistance: Current State of Knowledge and Clinical Implications—Toward a Better Diagnostic Framework and the Question of Its Disease Status

**DOI:** 10.3390/nu18162666

**Published:** 2026-08-14

**Authors:** Łukasz Rodzeń, Mateusz Rodzeń, Damian Dyńka, Dorota Łojko, Hanna Karakuła-Juchnowicz, Sebastian Kraszewski, Serafino Fazio, David Unwin, Benjamin Bikman

**Affiliations:** 1 Polish Society for Insulin Resistance Treatment (PTLI), 54-105 Wrocław, Poland; lukasz.rodzen@ptli.pl (Ł.R.); mateusz.rodzen@ptli.pl (M.R.); sebastian.kraszewski@pwr.edu.pl (S.K.); 2 Rodzen Brothers Foundation, 64-234 Wieleń, Poland; 3 Institute of Health Sciences, Faculty of Medical and Health Sciences, University of Siedlce, 08-110 Siedlce, Poland; 4 Department of Psychiatry, Poznan University of Medical Science, 60-572 Poznan, Poland; 5 Department of Psychiatry, Psychotherapy and Early Intervention, Medical University of Lublin, 20-439 Lublin, Poland; 6 Department of Biomedical Engineering, Faculty of Fundamental Problems of Technology, Wroclaw University of Science and Technology, 50-370 Wroclaw, Poland; 7 Department of Internal Medicine, School of Medicine, Federico II University, Via Sergio Pansini 5, 80131 Naples, Italy; 8 Faculty of Health Social Care and Medicine, Edge Hill University, Ormskirk L39 4QP, UK; 9 Department of Cell Biology and Physiology, Brigham Young University, 3052 LSB, Provo, UT 84602, USA

**Keywords:** insulin resistance, diagnosis of insulin resistance, hyperinsulinemia, type 2 diabetes, cardiovascular disease, cellular senescence, cancer, metabolic disease, metabolic prevention, low-carbohydrate diet

## Abstract

Insulin resistance (IR) represents one of the most pressing problems in contemporary public health. Its estimated global prevalence ranges from approximately 15.5% to over 61%, depending on the population studied, the diagnostic criteria applied, and the method used for its assessment. Despite the scale of the problem, IR remains underrecognized and lacks formal definition as a distinct disease entity, even as a growing number of clinicians and researchers worldwide describe it as such. Its asymptomatic or mildly symptomatic course allows it to remain undetected for years, during which it makes a significant contribution to the development of type 2 diabetes, cardiovascular disease (CVD) and metabolic dysfunction-associated steatotic liver disease (MASLD, formerly NAFLD), and has been increasingly linked to cellular senescence, certain cancers, neuropsychiatric disorders, and other metabolic conditions. The aim of this review was to summarize current knowledge on the pathophysiology, diagnosis, and clinical implications of insulin resistance, to discuss current challenges in its diagnosis, and to evaluate whether available scientific evidence supports its recognition as a distinct disease entity. This narrative review is based on clinical, epidemiological, and mechanistic data retrieved from PubMed and Google Scholar. Meta-analyses, systematic reviews, clinical and observational studies, clinical guidelines, and expert position statements were analyzed. Animal studies were excluded to maintain a focus on human public health implications. The diagnostic gold standard—the hyperinsulinemic-euglycemic clamp—was discussed, along with surrogate methods used in clinical practice (HOMA-IR, OGTT with insulin measurements, the TyG index, and the TG/HDL-C ratio). Factors potentially contributing to the pathogenesis of IR were examined, including hyperinsulinemia (HI), high-carbohydrate diets, inflammation, stress, and sleep disturbances, as well as conditions in which IR occurs physiologically. The findings indicate that current evidence supports the need for a clearer clinical and diagnostic framework for insulin resistance and suggest that its recognition as a distinct disease entity could facilitate earlier diagnosis, improve the standardization of clinical management, and enable earlier metabolic intervention. Given the steadily rising prevalence of metabolic disease, systemic efforts directed at the early identification and treatment of IR may be a key component of strategies aimed at reducing the population-level burden of metabolic disease and its negative consequences.

## 1. Introduction

Insulin resistance (IR) is a highly prevalent metabolic condition whose public health significance stems from its association with the development of numerous chronic diseases. Reported prevalence estimates vary substantially across populations, ranging from approximately 15.5–46.5% [1] to as high as 61.2% [2], depending on the population studied, diagnostic criteria applied, and assessment method. In the United States, insulin resistance has been reported in approximately 4 in 10 nondiabetic adults aged 18--44 years [3]. Despite this scale, IR remains an underappreciated clinical and public health challenge: its asymptomatic or mildly symptomatic course allows this metabolic disorder to persist for many years before manifesting as diabetes, cardiovascular disease, cancer, cognitive decline, or dementia, generating substantial, often unrecognized, health and societal costs along the way. IR is associated with the deterioration of metabolic health parameters and, furthermore, constitutes a key pathogenic factor in the development of numerous chronic diseases, as discussed in detail in Section 9.2. The scale of the problem is such that, were insulin resistance hypothetically classified as a distinct disease entity, it would most likely represent the most common disease in the world.

For these reasons, the implementation of systematic screening programs may be warranted, enabling the early identification of individuals with hyperinsulinemia and IR and the prompt initiation of preventive management [4]. This article incorporates the most recent scientific evidence—clinical, epidemiological, and mechanistic data, together with available health forecasts—integrating them into a coherent analysis of the problem. The aim of this review is to summarize current knowledge on the pathophysiology, diagnosis, and clinical implications of insulin resistance, to discuss current challenges in its diagnosis, and to evaluate whether the available scientific evidence supports its recognition as a distinct disease entity. By integrating current evidence, this review aims to contribute to a clearer clinical and diagnostic framework for insulin resistance and to highlight its potential implications for clinical practice and public health. The purpose of this review is not to propose a new formal definition of insulin resistance or to definitively determine its status as a distinct disease entity, but rather to critically evaluate the available evidence, identify current gaps in knowledge and clinical practice, and provide a foundation for future research and expert consensus. Unless these gaps are addressed at the level of fundamental pathophysiological mechanisms and clinical recognition, healthcare systems may continue to struggle to achieve meaningful reductions in the burden of metabolic disease—a challenge already reflected in the persistent rise in the incidence of these disorders and in projections for the coming years. Throughout this review, the term ‘metabolic disease’ is used broadly to denote the range of chronic conditions associated with metabolic dysfunction—including type 2 diabetes, cardiovascular disease, and MASLD—whereas ‘metabolic syndrome’ refers to the specific, formally defined clinical entity discussed in Section 9.2.3. A stronger public health focus on the early detection and management of IR/hyperinsulinemia could contribute meaningfully to improving population health.

## 2. Methodology

This publication takes the form of a narrative review, a format selected given the breadth of the subject matter and the need for a comprehensive treatment of issues spanning insulin resistance, metabolic disease, nutrition, and public health. The analysis draws on a range of scientific sources, including meta-analyses, systematic reviews, clinical studies, mechanistic studies, observational studies, epidemiological data, clinical guidelines, and expert position statements. Given the nature and heterogeneity of the available data, their synthesis within the framework of a classical systematic review was not feasible. Animal studies were deliberately excluded from this article, as its aim is to highlight the practical implications for human public health. The literature search was conducted primarily using the PubMed and Google Scholar databases. Combinations of keywords relating to IR, hyperinsulinemia, diagnosis, epidemiology, treatment, and public health were used, with the search strategy tailored to the subject matter of each individual section.

## 3. Insulin Resistance: A Biological and Clinical Perspective

As a biological phenomenon, IR is relatively well characterized in the scientific literature, reflecting decades of intensive research into its molecular, metabolic, and endocrine mechanisms. For the purposes of this review, however, it is useful to distinguish between two complementary perspectives: a biological definition, referring to the mechanisms underlying reduced tissue sensitivity to insulin, and a clinical perspective, which concerns the diagnostic identification and assessment of this condition in medical practice.

### 3.1. Biological Definition

Insulin resistance (IR) is defined as an impaired biological response of target tissues (principally the liver, muscle tissue, and adipose tissue) to insulin action. In skeletal muscle and adipose tissue, this is reflected primarily in impaired GLUT4-mediated glucose uptake, whereas in the liver it manifests chiefly as impaired insulin-mediated suppression of hepatic glucose production. Notably, tissues exhibiting reduced GLUT4-mediated glucose uptake may simultaneously retain or even show heightened sensitivity to insulin’s growth-promoting MAPK signaling, a distinction often overlooked in IR research [5,6,7]. This impairment leads to reduced glucose uptake by target tissues and an attempt at a compensatory increase in insulin secretion by pancreatic β-cells (resulting in hyperinsulinemia), trying to maintain glucose levels in the normal range. Hyperinsulinemia is increasingly recognized as more than a compensatory response, with important pathophysiological and clinical consequences of its own. The roles of both IR and hyperinsulinemia in the development of metabolic disorders and diseases [8,9] are explored further in Section 9.2, with the underlying pathomechanisms of IR discussed in Section 5.

### 3.2. Clinical Perspective

The clinical perspective concerns the methods used to identify and assess IR in diagnostic practice. In the absence of a simple, standardized diagnostic test suitable for routine clinical use, the diagnosis of IR most commonly relies on surrogate measures (e.g., HOMA-IR, the oral glucose tolerance test with insulin measurement, the TyG index, and the TG/HDL-C ratio) [8], the advantages and limitations of which are discussed in Section 6.

Although the biological definition of IR is relatively well established, the central challenge lies in its clinical and diagnostic operationalization. The absence of universally accepted diagnostic criteria remains a major obstacle to recognizing IR as an independent disease entity.

## 4. Physiological Insulin Resistance

Insulin resistance is not always a pathological phenomenon, nor is it solely a consequence of adverse lifestyle-related factors. At certain stages of human life, it can serve as a physiological metabolic adaptation. Among the best-described examples are puberty and pregnancy, during which a transient reduction in tissue sensitivity to insulin forms part of normal hormonal and metabolic processes. Particular attention should also be given to the physiological IR that occurs transiently in individuals following low-carbohydrate dietary patterns, as discussed in Section 4.3.

### 4.1. Physiological Insulin Resistance During Puberty

Despite the rising prevalence of pathological insulin resistance among children and adolescents [10], puberty is also characterized by a physiological and transient reduction in tissue sensitivity to insulin, with reductions reported to reach approximately 50% [11]. Both prepubertal children and adults have been shown to have greater insulin sensitivity than children undergoing puberty. Insulin resistance is often most pronounced in mid-puberty, corresponding to Tanner stages T3/T4, with insulin sensitivity typically recovering by stage T5, although this pattern is not universal [12].

This transient reduction in insulin sensitivity is thought to result primarily from the action of growth hormone, which exerts insulin-antagonistic effects. It is typically accompanied by compensatory hyperinsulinemia, which helps maintain normal glucose homeostasis while supporting the anabolic processes characteristic of this period of intensive growth [11,12]. Mechanistically, growth hormone stimulates lipolysis, increasing circulating free fatty acids, which impair insulin signaling in skeletal muscle through IRS-1/PI3K-Akt pathways. In addition, experimental evidence suggests that growth hormone may increase expression of the p85 regulatory subunit of PI3K, representing a potential post-receptor mechanism contributing to insulin resistance [13].

### 4.2. Physiological Insulin Resistance During Pregnancy

In women, pregnancy represents another physiological state in which insulin resistance characteristically occurs. During pregnancy, insulin sensitivity decreases by approximately 50–60% [14]. In normal pregnancy, a gradual increase in IR/HI is observed, driven by placental hormones, including human placental lactogen, estrogens, progesterone, human chorionic gonadotropin, growth hormone, and prolactin, as well as cortisol and inflammatory mediators, including adipokines such as leptin and TNF-α. This adaptation is accompanied by compensatory changes in pancreatic β-cell function and insulin secretion, allowing maternal glycemia to be maintained while ensuring an adequate glucose supply for the developing fetus [15]. At the molecular level, TNF-α derived from both the placenta and maternal tissues promotes serine phosphorylation of IRS-1, disrupting downstream PI3K/Akt signaling and impairing GLUT4 translocation in maternal skeletal muscle and adipose tissue [16,17], while other placental hormones, including human placental lactogen, cortisol, and progesterone, contribute through complementary mechanisms that are less completely characterized. When IR/HI becomes excessive, for example in the presence of pre-existing pathological IR, β-cell compensation may become insufficient, leading to elevated glycemia and potentially resulting in gestational diabetes [18]. Even among women without diabetes, the risk of congenital heart defects in offspring has been shown to increase by 8% for every 10 mg/dL increase in maternal glucose concentration above reference values in early pregnancy [19]. This observation underscores the importance of optimizing metabolic status before a planned pregnancy, including the reduction in IR and hyperinsulinemia, as a potential strategy for reducing the risk of both maternal and fetal complications.

### 4.3. Physiological Insulin Resistance in Individuals Following Low-Carbohydrate Dietary Models

In individuals following a very low-carbohydrate diet or undergoing prolonged fasting, a metabolic adaptation known as “glucose sparing” occurs—a key survival mechanism that preserves glucose for tissues with an obligate requirement, particularly the brain [20]. Although ketone bodies substantially reduce cerebral glucose requirements, they cannot fully replace glucose. Beyond serving as an energy substrate, glucose is required for the pentose phosphate pathway, which generates NADPH for glutathione-dependent antioxidant defense, and acts as a carbon precursor for the synthesis of neurotransmitters such as glutamate, GABA, and acetylcholine [20]. Ketone bodies can reduce glucose utilization for the brain’s energy needs while helping preserve glucose availability for other biological functions, a mechanism that may contribute to the therapeutic effects of ketogenic diets in conditions such as epilepsy [21]. Increased oxidation of fatty acids and ketone bodies limits glucose utilization by skeletal muscle, thereby preserving its availability for tissues more dependent upon it. This mechanism is partly explained by the Randle cycle, which describes the competition between fatty acid and glucose oxidation [20]. It is therefore unsurprising that individuals restricting carbohydrate intake show significantly elevated glucose and insulin responses during an oral glucose tolerance test (OGTT); however, this does not necessarily indicate a pathological response, but rather reflects the body’s adaptive mechanisms [22]. This phenomenon has been described in the scientific literature for nearly a century. In a classic study conducted in 1929, two men following an exclusively meat-based diet for one year were evaluated. At the end of the intervention, an oral glucose tolerance test (OGTT) revealed an abnormal glycemic response. However, after returning to a carbohydrate-containing diet and repeating the OGTT 2–4 weeks later, results were normal [23]. The observed impairment in glucose tolerance was attributed to metabolic adaptation to prolonged carbohydrate restriction and reduced insulin secretion. The rapid normalization of glycemic response following carbohydrate reintroduction indicates that this phenomenon represented a reversible physiological adaptation rather than a permanent metabolic disorder. It should also be noted that individuals with a prior history of hyperinsulinemia who subsequently adopt very low-carbohydrate diets may exhibit different, and potentially more pronounced, OGTT responses compared with metabolically healthy individuals with long-term dietary adaptation, in whom insulin sensitivity may in fact be preserved or enhanced [24,25,26].

For this reason, performing an OGTT in individuals following very low-carbohydrate dietary patterns without appropriate dietary preparation is not recommended, as antecedent carbohydrate restriction may yield false-positive results, cause patient anxiety, or potentially lead to misdiagnosis of diabetes. In individuals who have adopted such dietary patterns to manage pre-existing metabolic conditions, deliberate carbohydrate reintroduction for testing purposes also raises ethical considerations that warrant clinical judgment on a case-by-case basis. If such an individual nevertheless wishes to undergo, or requires, an OGTT, appropriate preparation is essential. For example, one publication [27] suggested that, to avoid a distorted OGTT result, carbohydrate intake should be increased to a minimum of 150 g per day for at least 3 days prior to testing, ideally distributed across 3 meals, with each meal—particularly the evening meal preceding the fasting test—providing a minimum of 50 g of carbohydrates. However, there is growing evidence that this preparation period may be insufficient. One study [28] demonstrated that the standard recommended 3-day preparation period with a carbohydrate intake of ≥150 g/day may be inadequate for individuals chronically following a low-carbohydrate diet, creating a genuine risk of false-positive diabetes diagnosis.

It should be emphasized that, when interpreting carbohydrate metabolism test results in individuals following low-carbohydrate diets, a detailed dietary history accounting for current carbohydrate intake is of critical importance. This helps minimize the risk of misinterpretation and the overdiagnosis of glucose metabolism disorders in metabolically healthy individuals. In this patient population, diagnosis should not rely solely on oral glucose tolerance test (OGTT) results or the insulin curve, but should also take into account glycated hemoglobin (HbA1c) concentration—bearing in mind its potential unreliability in individuals with altered erythrocyte turnover—dietary context, and a comprehensive assessment of metabolic status.

**Figure 1:**
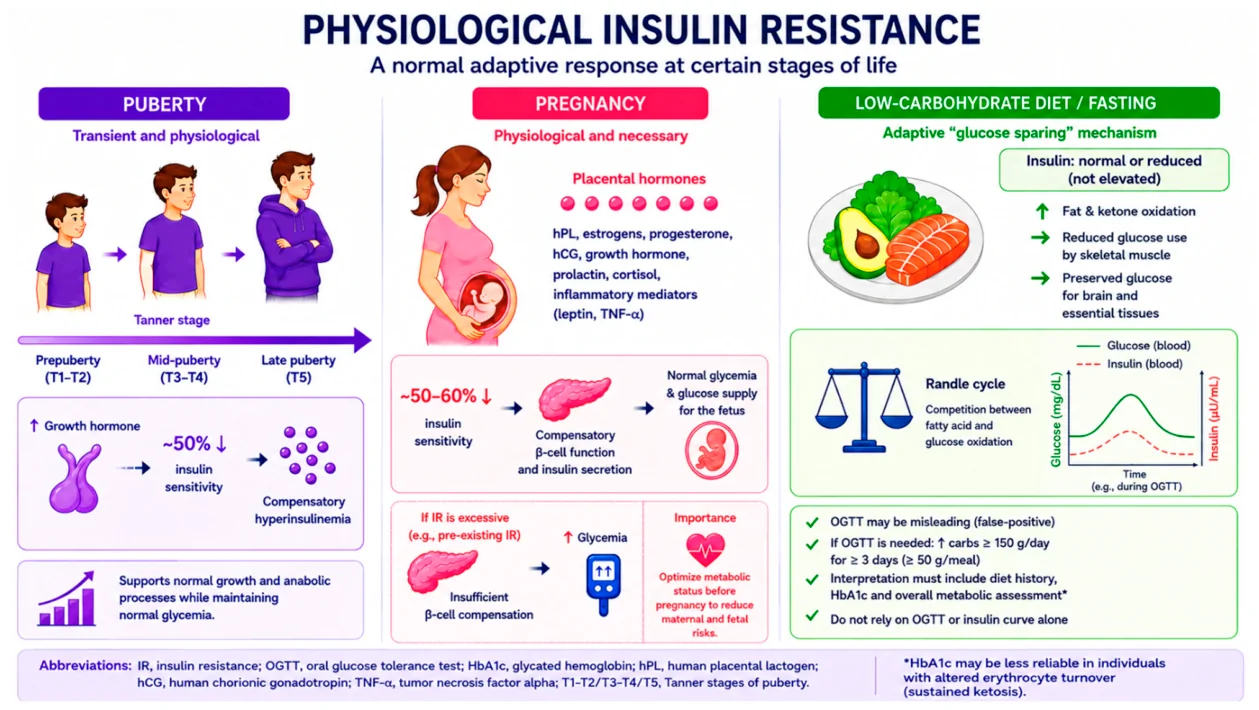
Physiological insulin resistance.

The examples presented here demonstrate that reduced insulin sensitivity is not invariably a pathological phenomenon—in certain physiological states, it forms part of a normal adaptive response that enables the maintenance of energy homeostasis or meets heightened metabolic demands. The diagnosis of pathological insulin resistance should therefore always take into account the clinical context, patient age, physiological state, and dietary pattern. Physiological insulin resistance is illustrated in Figure 1.

## 5. Mechanisms Underlying the Development of Insulin Resistance

Insulin resistance develops through the interaction of multiple environmental, metabolic, and hormonal factors. Understanding its underlying mechanisms, however, requires consideration of a fundamental question of causality: is insulin resistance the primary metabolic disturbance that leads to hyperinsulinemia, or does hyperinsulinemia precede and drive the development of insulin resistance? The answer to this question has important implications for both diagnosis and treatment.

### 5.1. Factors Initiating and Sustaining Insulin Dysregulation

A range of environmental and metabolic factors have a documented influence on insulin secretion and tissue sensitivity to its action. Their common denominator is the capacity to induce or amplify hyperinsulinemia, regardless of which pathogenetic model is considered predominant.

#### 5.1.1. Hyperinsulinemia as a Contributor to the Initiation and Maintenance of IR

**Figure 2:**
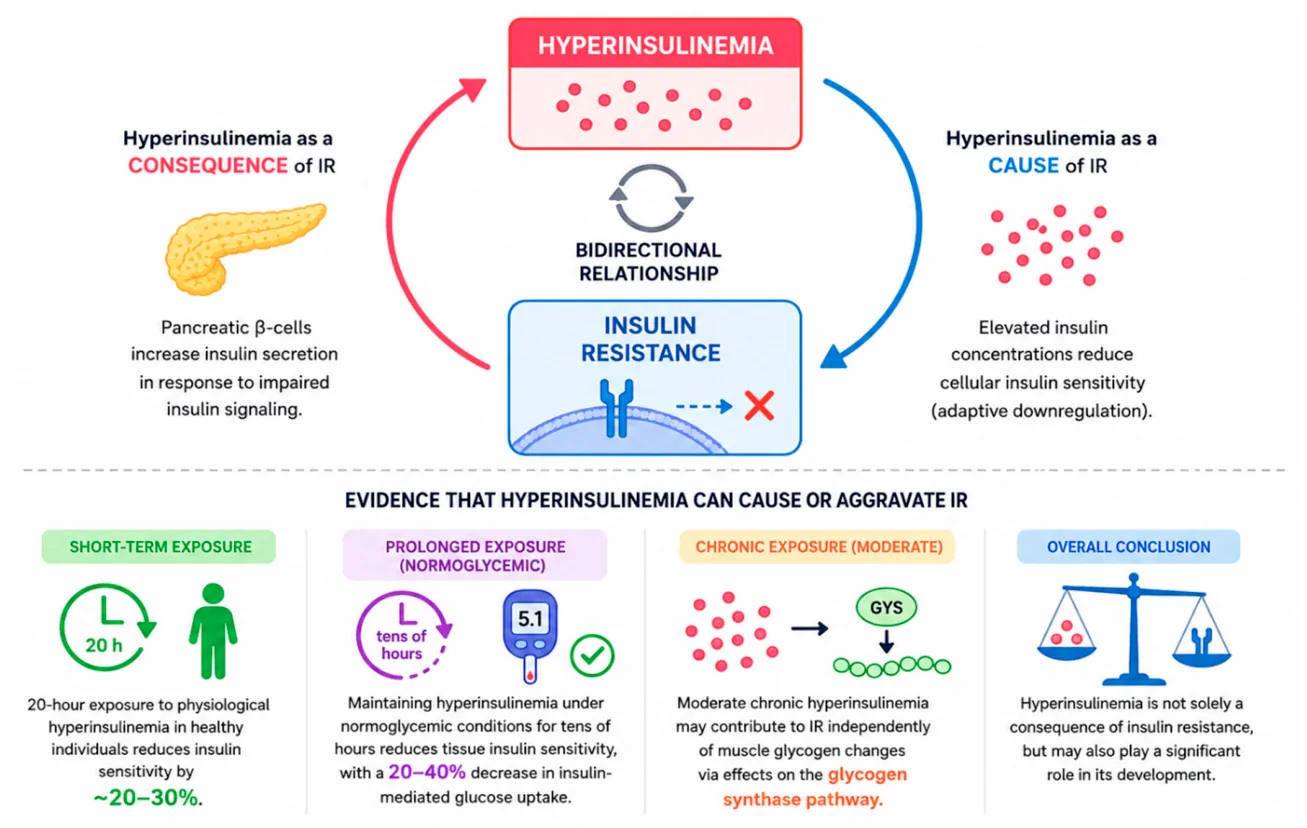
Hyperinsulinemia as a Contributor to the Initiation and Maintenance of IR.

One of the conceptually most important factors is hyperinsulinemia itself. For decades, the prevailing model held that IR was the primary phenomenon, with hyperinsulinemia representing primarily a compensatory response of pancreatic β-cells to impaired insulin signaling [8]. However, available data increasingly point to a bidirectional relationship: hyperinsulinemia may be both a consequence and a cause of IR. Short-term exposure to elevated insulin concentrations can lead to an adaptive reduction in cellular insulin sensitivity. This phenomenon was documented more than four decades ago, when a 20 h exposure to physiological hyperinsulinemia in healthy individuals produced a significant decline in insulin sensitivity of approximately 20–30% [29]. Subsequent studies confirmed that maintaining hyperinsulinemia under normoglycemic conditions for tens of hours results in a substantial reduction in tissue insulin sensitivity, corresponding to a 20–40% decrease in insulin-mediated glucose uptake, even in healthy individuals [30]. Moreover, moderate chronic hyperinsulinemia may contribute directly to IR, independently of changes in muscle glycogen concentration, through its effects on the glycogen synthase pathway [31]. Taken together, these findings suggest that hyperinsulinemia is not solely a consequence of insulin resistance, but may also play a significant role in its development. This relationship is discussed in greater detail in Section 5.2. Hyperinsulinemia as a contributor to the initiation and maintenance of IR is illustrated in Figure 2. Chronically elevated insulin concentrations promote ubiquitin-mediated degradation and downregulation of IRS proteins while activating negative-feedback serine kinases, including JNK, that phosphorylate IRS-1 and impair downstream insulin signaling [32].

#### 5.1.2. Stress, Sleep Disturbances, and Neuroendocrine Activation

**Figure 3:**
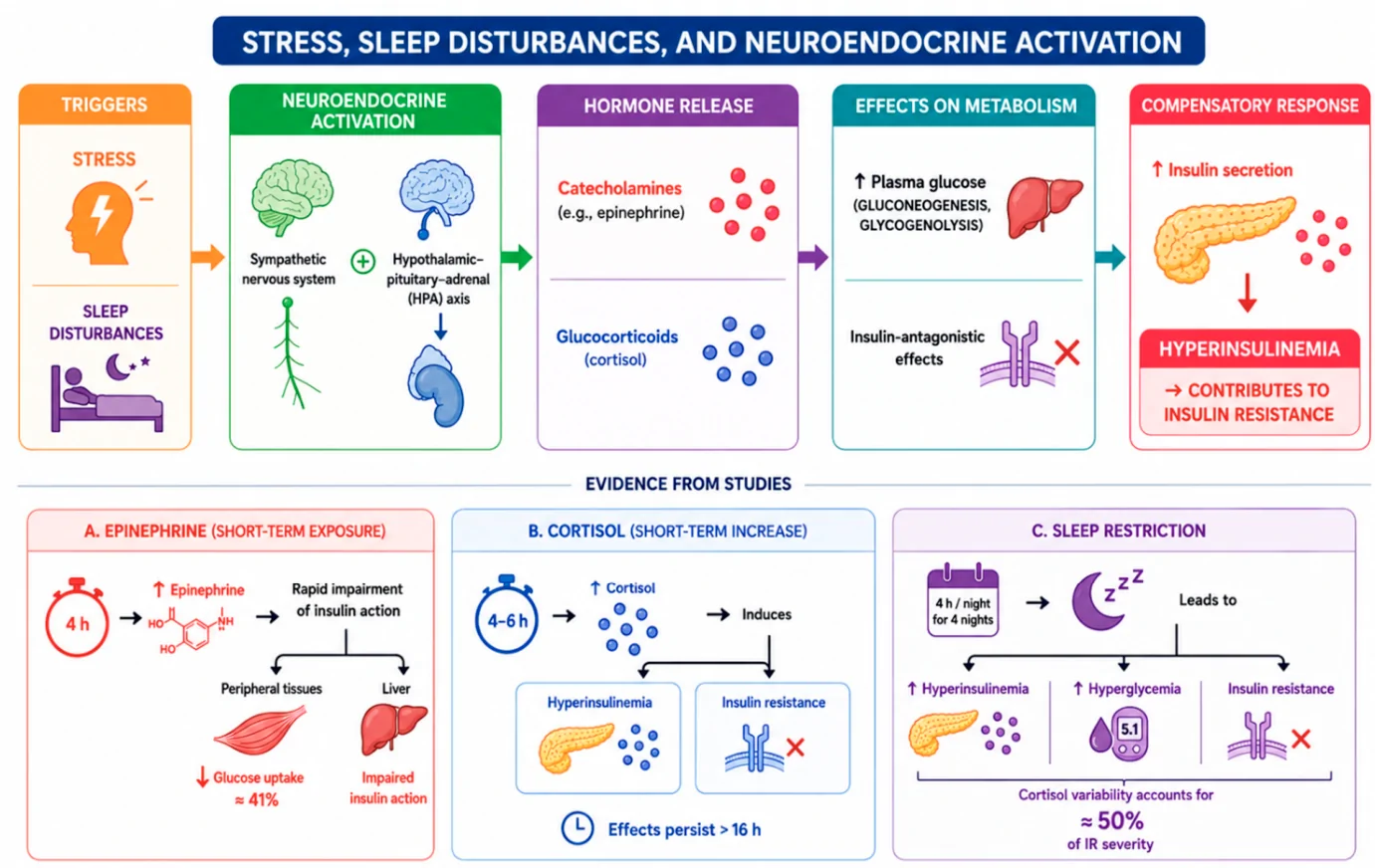
Stress, sleep disturbances, and neuroendocrine activation as factors exacerbating insulin resistance.

Stress, mediated through activation of the sympathetic nervous system and the hypothalamic–pituitary–adrenal axis, leads to increased secretion of catecholamines (including epinephrine) and glucocorticoids (cortisol). These hormones amplify processes that raise plasma glucose concentrations (gluconeogenesis, glycogenolysis) and exert insulin-antagonistic effects [33,34], which in turn drive a compensatory increase in insulin secretion. Even a 4 h exposure to elevated epinephrine concentrations has been shown to produce a rapid impairment of insulin action in both peripheral tissues (a reduction in glucose uptake of approximately 41%) and the liver [35]. Similarly, an increase in serum cortisol can induce hyperinsulinemia and IR within 4–6 h, with these effects persisting for more than 16 h [36]. Sleep disturbances reproduce this same mechanism: restricting sleep to 4 h per night for 4 consecutive nights led to the development of hyperinsulinemia, hyperglycemia, and IR, with cortisol variability accounting for approximately 50% of the IR severity observed under conditions of sleep deprivation [37]. Experimentally restricted sleep reduces insulin-stimulated Akt phosphorylation in human adipocytes, producing tissue-level insulin resistance [38]. Similarly, glucocorticoids promote inhibitory serine phosphorylation of IRS-1 while reducing its tyrosine phosphorylation, thereby attenuating downstream Akt signaling [34]. These data suggest that sleep disturbances may represent a significant factor promoting the development of hyperinsulinemia and secondary insulin resistance. Stress, sleep disturbances, and neuroendocrine activation as factors exacerbating IR are illustrated in Figure 3.

#### 5.1.3. Inflammation

Inflammation, whether triggered by infection, trauma, or autoimmune disease, is another factor capable of rapidly impairing insulin sensitivity. In experimental infection models, glucose disposal has been shown to decrease by as much as 52–59%, with the magnitude of IR/HI comparable to that predicted for an 84-year-old individual or a person with class II obesity [39]. Mechanistically, pro-inflammatory cytokines such as TNF-α promote serine phosphorylation of IRS-1, disrupting downstream PI3K/Akt signaling and thereby impairing GLUT4 translocation [17].

IR can develop rapidly during infection or systemic inflammation, as demonstrated 420 min after administration of bacterial lipopolysaccharide, an experimental model of systemic inflammatory response [40]. Recent publications further support the role of inflammation in the rapid induction of IR [41,42]. Importantly, inflammation-induced IR is not limited to infectious or autoimmune stimuli. Certain dietary components may also contribute to inflammation-driven metabolic dysfunction. For example, a recent randomized double-blind cross-over trial suggested that carrageenan, a widely used food additive, may impair insulin sensitivity and promote low-grade gut-derived inflammation in overweight individuals [43].

#### 5.1.4. High-Carbohydrate Diets, Hyperinsulinemia, and Hyperphagia

Diets rich in high-glycemic-index or high-glycemic-load carbohydrates are among the best-described dietary factors associated with postprandial hyperinsulinemia. Rapidly absorbed carbohydrates cause a sharp rise in postprandial glycemia, eliciting a robust insulin response. According to the carbohydrate-insulin model of obesity (CIM), postprandial hyperinsulinemia preferentially directs energy toward storage in adipocytes, reducing its availability as fuel for other tissues. The resulting relative energy deficit in peripheral tissues may stimulate central hunger mechanisms and promote hyperphagia [44,45]. Under this framework, overeating is interpreted less as the primary initiating cause of obesity and hyperinsulinemia and more as a downstream response to hormonally mediated partitioning of energy toward adipose tissue, which may subsequently promote excess food intake. From an evolutionary physiology perspective, this mechanism may have represented an adaptation that enhanced survival in environments characterized by intermittent food availability. Under this hypothesis, postprandial hyperinsulinemia may have favored the efficient storage of surplus energy as adipose tissue, building reserves that could be drawn upon during periods of scarcity. At the same time, reduced availability of circulating energy substrates to peripheral tissues may have activated mechanisms that heighten appetite, which, under conditions of irregular food supply, could have favored maximizing energy intake whenever food was available. While potentially advantageous under conditions of cyclical food scarcity, this same mechanism, in a modern environment characterized by the constant availability of highly processed food, may instead promote chronic hyperinsulinemia, excessive energy storage, and the development of obesity and insulin resistance [46,47,48].

**Figure 4:**
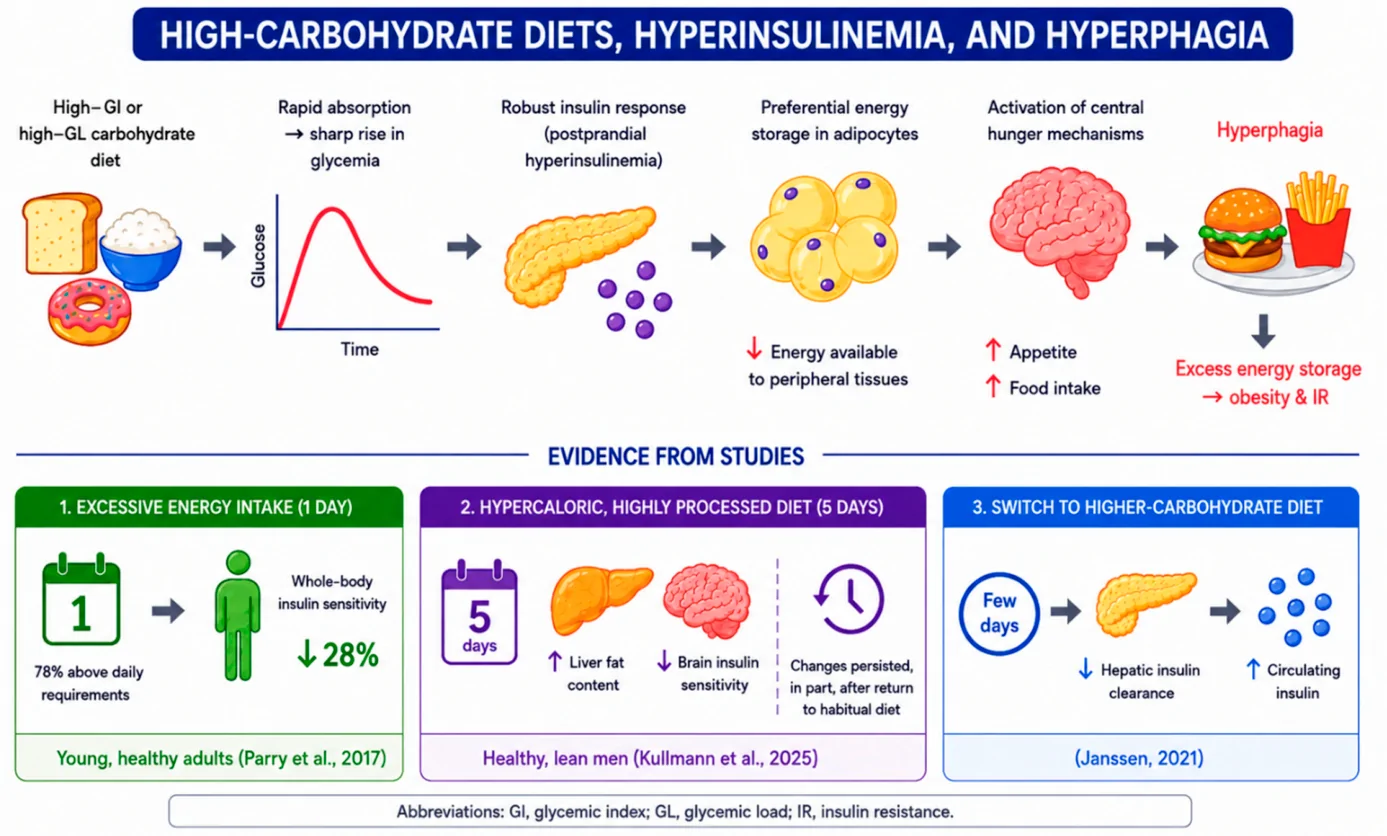
The impact of high-carbohydrate diets, hyperinsulinemia, and hyperphagia on insulin resistance [49,50,51].

A single day of excessive energy intake, corresponding to 78% above daily requirements, has been shown to reduce whole-body insulin sensitivity by 28% in young, healthy adults [49]. In another study involving healthy, lean men, a five-day intervention based on a hypercaloric, highly processed diet led to increased liver fat content and reduced brain insulin sensitivity, despite the absence of significant changes in body weight. These changes persisted, in part, even after a return to habitual dietary patterns, suggesting that impairments in central nervous system insulin sensitivity may develop rapidly and precede the onset of obesity and overt metabolic disturbances [50]. Additional evidence suggesting that dietary carbohydrate may influence circulating insulin independently of weight gain comes from the observation that hepatic insulin clearance can decline within a few days of switching to a higher-carbohydrate diet [51], potentially leading to higher systemic insulin concentrations before any meaningful change in body weight occurs. The impact of high-carbohydrate diets, hyperinsulinemia, and hyperphagia on insulin resistance is illustrated in Figure 4.

### 5.2. Two Models of Pathogenesis: Hyperinsulinemia Versus Insulin Resistance—Which Comes First?

A central question in contemporary research on the pathogenesis of insulin resistance concerns the direction of causality between hyperinsulinemia and insulin resistance. The factors described above may induce insulin dysregulation through two conceptually distinct, although potentially overlapping, pathways corresponding to two pathogenetic models described in the literature. The classical model holds that IR is the primary abnormality: peripheral tissues, including skeletal muscle, liver, and adipose tissue, lose sensitivity to insulin, prompting pancreatic β-cells to compensate by increasing insulin secretion, which results in hyperinsulinemia. When β-cell compensatory capacity becomes insufficient, overt hyperglycemia and type 2 diabetes may ensue [8].

An alternative model, which has received increasing attention in recent years but remains an area of active investigation, proposes a reversed or partially reversed sequence: chronic insulin hypersecretion, driven by genetic, epigenetic, and environmental factors, including high-carbohydrate diets, stress, and sleep disturbances, may represent an upstream event that contributes to the subsequent development of IR [51,52,53]. Within this framework, tissue insulin resistance has been proposed to function, at least in part, as a physiological protective adaptation: an attempt to shield critical tissues from the metabolic stress imposed by chronic exposure to elevated insulin concentrations [9,54].

These two models are not necessarily mutually exclusive. Current evidence suggests that both mechanisms may contribute to the development of IR, with their relative importance likely varying across individuals and stages of metabolic dysfunction. In clinical practice, hyperinsulinemia and IR likely interact in a self-perpetuating vicious cycle. Nevertheless, the possibility that hyperinsulinemia may act not only as a consequence but also as an initiating or amplifying factor has important therapeutic implications. Interventions that lower insulin demand and thus concentration and exposure, including low-carbohydrate diets, caloric restriction, and enhancement of hepatic insulin clearance, may have therapeutic relevance not only in type 2 diabetes and obesity, but also in other insulin-associated chronic diseases, including cardiovascular disease and certain cancers [51].

### 5.3. Obesity as a Possible Consequence of Hyperinsulinemia: Revisiting the Direction of Causality?

Adipocyte hypertrophy has traditionally been described as the initiating event in a cascade leading to systemic insulin resistance (IR). However, an alternative causal sequence has also been proposed, in which chronic hyperinsulinemia may contribute to the development of obesity, while obesity—particularly visceral obesity—further exacerbates IR, creating a self-perpetuating cycle, as outlined below.

Insulin is the dominant anabolic hormone of adipose tissue: it stimulates de novo lipogenesis, suppresses lipolysis, increases the uptake of fatty acids from circulating lipoproteins via activation of lipoprotein lipase, and, over the longer term, drives adipogenesis through the induction of key transcription factors (C/EBPα, PPARγ) [55,56]. States of chronic hyperinsulinemia—such as insulin-secreting tumors or the initiation of insulin therapy in diabetes—are commonly associated with weight gain [57,58]. Conversely, drugs that attenuate hyperinsulinemia (e.g., diazoxide, octreotide) lead to weight loss and improved insulin sensitivity [59,60,61].

The carbohydrate-insulin model (CIM) represents one proposed explanation for the pathogenesis of obesity and remains the subject of ongoing scientific debate. According to this model, diets rich in high-glycemic-index carbohydrates promote postprandial hyperinsulinemia, which preferentially directs calories toward adipose tissue rather than oxidation in lean tissues, thereby facilitating fat accumulation [44,45]. Within this framework, increased energy intake is considered secondary to hormonal and metabolic changes, particularly hyperinsulinemia, which may enhance energy storage, increase hunger, and promote a positive energy balance.

Regardless of the underlying model, progressive adipocyte hypertrophy is associated with metabolic dysfunction and reduced insulin sensitivity [62,63,64]. Enlarged adipocytes exhibit reduced GLUT4 expression and impaired insulin signaling, limiting further substrate uptake and potentially serving as a protective mechanism against excessive cellular overload [65,66,67]. Consequently, adipose tissue becomes resistant to insulin’s antilipolytic action, resulting in increased free fatty acid release despite hyperinsulinemia. In metabolic syndrome, adipose tissue sensitivity to insulin-mediated suppression of lipolysis is reduced approximately tenfold [68]. Elevated circulating free fatty acids, together with hyperinsulinemia, promote ectopic lipid deposition in the liver, skeletal muscle, and pancreas. Excess insulin also impairs fatty acid oxidation in these tissues, contributing to lipotoxicity and further deterioration of insulin sensitivity [69,70,71,72].

As adipocytes undergo hypertrophy, relative adipose tissue hypoxia develops because angiogenesis fails to keep pace with tissue expansion. This activates hypoxia-dependent pathways, including HIF-1α, leading to increased production of proinflammatory cytokines (e.g., TNF-α) and angiogenic factors such as VEGF. Consequently, adipose tissue acquires a proinflammatory phenotype, contributing to chronic low-grade systemic inflammation [62,73,74,75,76]. Importantly, metabolic risk depends less on the total amount of adipose tissue than on its distribution. Visceral adipose tissue is highly metabolically active and strongly associated with insulin resistance, inflammation, and an increased risk of type 2 diabetes and cardiovascular disease [77,78].

Adipocyte hypertrophy requires both a positive energy balance and sufficient insulin exposure. Energy surplus alone, in the setting of absolute insulin deficiency, does not result in effective fat storage. This is illustrated by patients with type 1 diabetes, who may lose weight despite adequate or increased caloric intake [79], and even more clearly by individuals with intentional insulin omission for weight control (diabulimia), who may fail to gain weight despite consuming high-calorie diets [80,81,82].

Conversely, hyperinsulinemia alone, in the absence of an energy surplus, does not increase adipose tissue mass. Nevertheless, chronically elevated insulin concentrations may promote energy storage by suppressing lipolysis, stimulating triglyceride synthesis, and shifting metabolism toward an anabolic state. These observations suggest that hyperinsulinemia may actively contribute to adipocyte hypertrophy when excess energy is available. According to the carbohydrate-insulin model (CIM), the principal distinction from the traditional energy balance model lies in the proposed mechanisms leading to a positive energy balance, rather than in the requirement for a positive energy balance itself [44].

## 6. Diagnostic Assessment of Insulin Resistance

Given the growing prevalence of IR/HI, sometimes described as a “silent pandemic”, broader use of available assessment tools appears warranted. Although no single official diagnostic criterion has been universally adopted, several methods and surrogate indices are available that may facilitate the identification of individuals likely to have IR. All diagnostic methods and surrogate indices discussed in this section are summarized in Table 1, presented at the end of the section.

### 6.1. Euglycemic Hyperinsulinemic Clamp (EHC): The Gold Standard

The euglycemic-hyperinsulinemic clamp (EHC) represents the gold standard for the diagnosis of IR [83,84]. It should be noted that the EHC provides a single, controlled measurement of insulin-mediated glucose disposal and does not capture longitudinal or free-living patterns of insulin exposure over time. The procedure involves the infusion of insulin to achieve a constant state of hyperinsulinemia, combined with a simultaneous glucose infusion designed to maintain euglycemia (~90–100 mg/dL). At steady state, the rate of glucose infusion corresponds to tissue glucose uptake and is expressed as the M-value (mg/kg/min). An M-value below 5 mg/kg/min is generally regarded as the optimal indicator of IR and represents the accepted cutoff [85]. Although the EHC is the most reliable and precise measure available, its cost, time demands, and requirement for specialized research infrastructure mean that its use is largely confined to research settings. For this reason, several more feasible surrogate indices have been developed and are used in clinical and epidemiological settings, as discussed in the following sections.

### 6.2. Homeostatic Model Assessment of Insulin Resistance (HOMA-IR)

The Homeostatic Model Assessment of Insulin Resistance (HOMA-IR) is one of the most widely used, simple, and practical surrogate indices for assessing IR in clinical practice, calculated as (fasting glucose × fasting insulin)/22.5 (for mmol/L) or/405 (for mg/dL) [86]. Although a cutoff value of 2.5 (in adults) and 3.6 (in children and adolescents) is frequently adopted [3], discrepancies across studies can be substantial. For example, a study of the Brazilian population identified a cutoff of 2.35 [87], while other studies reported cutoffs of 1.878 in Qatar [88], 1.70 for men and 1.78 for women in China [89], and 2.4 in Bangladeshi children [90]. Across numerous other studies, including populations from the United States, Sweden, France, Brazil, Japan, Iran, and Portugal, reported cutoff values range from 1.7 to 3.875 [91]. A study conducted in the Czech population identified a HOMA-IR range of 1.82 to 3.63 as a potential borderline interval for prediabetes [92]. These findings illustrate substantial variation in proposed HOMA-IR cutoff values across populations, reflecting differences in ethnic background, age distribution, prevalence of obesity, laboratory methodology, and the diagnostic criteria applied to define insulin resistance. As a result, no single, universally accepted cutoff value for HOMA-IR currently exists.

### 6.3. Homeostasis Model Assessment 2 (HOMA-2)

Homeostasis Model Assessment 2 (HOMA-2) is an updated mathematical model for assessing glucose-insulin homeostasis, representing a refinement of the original HOMA-1 model [93]. Developed by the Diabetes Trials Unit, it accounts for nonlinear relationships within the model, which may improve accuracy compared with HOMA-1. It allows for the assessment of pancreatic β-cell function (HOMA-2-%B), the degree of insulin resistance (HOMA-2-IR), and insulin sensitivity (HOMA-2-%S), with HOMA-2-%S representing the reciprocal of HOMA-2-IR [93,94]. One study reported that HOMA-2 was a more reliable predictor of type 2 diabetes development than the original HOMA-1 model [95], whereas another found that HOMA-1-IR demonstrated higher sensitivity and specificity than HOMA-2-IR in distinguishing individuals with type 2 diabetes from healthy controls [96]. The absence of clearly defined cutoff values for this index means that its clinical use remains limited, and it does not currently constitute a standardized diagnostic criterion for IR.

### 6.4. Fasting Insulin

Fasting insulin concentration is one of the simplest and most accessible surrogate markers of IR. The most recent scientific report of the Dietary Guidelines Advisory Committee for 2025–2030 highlights fasting insulin as a readily available marker for detecting IR, useful across diverse populations, including individuals with obesity, and as an important component of both clinical assessment and scientific research, alongside other surrogate indices [9,97,98]. Fasting insulin concentration has been shown in some studies to correlate strongly with values obtained using the EHC (the gold standard for assessing insulin sensitivity [99,100]), while remaining straightforward to measure in clinical practice, a combination that has long made it one of the most practical approaches for the screening assessment of IR [101]. Given the role of IR as a risk factor for dozens of chronic diseases, fasting insulin measurement represents a valuable and currently underused screening tool.

The euglycemic-hyperinsulinemic clamp remains the reference method; however, in clinical practice it is too complex and costly for widespread screening use. Fasting insulin may partially fulfill this role, provided its limitations are understood: no single universal diagnostic threshold exists, as results depend on the population studied, age, degree of obesity, sex, and the laboratory method employed.

Discrepancies between studies are substantial. For example, in a large Iranian population, a fasting insulin concentration of 2–12 μU/mL characterized 95% of healthy individuals of both sexes [102], whereas in a population from Mexico City, a cutoff of 14.38 μU/mL was adopted [103]. In a Korean population, a value of 12.94 μU/mL indicated IR, while a value as low as 10.57 μU/mL was already associated with an increased risk of metabolic syndrome [104]. Other data suggest a threshold for insulin resistance as low as ≥12.2 μU/mL in adults—particularly in the presence of normal fasting glycemia—as one of the cutoff values repeatedly cited in the literature for a simple screening test, demonstrating good sensitivity and specificity even in individuals with obesity [97,98]. A fasting insulin concentration below 8 μU/mL in men and below 10 μU/mL in women appears to represent a reasonable target range for metabolically healthy individuals, corresponding to values derived from the distribution of insulin concentrations observed in a metabolically healthy population: 4.6 ± 1.8 mU/L in men and 5.6 ± 2.3 mU/L in women [105]. The same study found that mean fasting insulin concentrations among individuals with advanced metabolic syndrome ranged from 15.4 to 15.6 mU/L; values exceeding 15 mU/L can therefore reasonably be regarded as thresholds suggestive of advanced metabolic dysfunction.

A distinction should be drawn between the laboratory reference range and the metabolically optimal range. For example, an upper laboratory reference limit of 13.14 μU/mL has been reported in a large laboratory analysis [106]. However, the upper limit of a laboratory reference range should not be interpreted as a metabolically low-risk value, but rather as a statistical boundary derived from the laboratory population. Given the variability across studies and populations, the proposed thresholds should be regarded as operational values for screening and risk stratification rather than as absolute, standalone diagnostic criteria for disease. Nevertheless, fasting insulin is recommended as a surrogate measure of insulin resistance, alongside HOMA-IR and QUICKI [104]. Its interpretation, however, must always take into account the patient’s clinical and population context.

### 6.5. Oral Glucose Tolerance Test (OGTT) with Insulin Measurement

Fasting insulin and glucose concentrations (used, for example, to calculate HOMA-IR) do not capture the dynamic glycemic and insulinemic response to a glucose load—information that is highly relevant to the assessment of IR. For this purpose, the oral glucose tolerance test (OGTT) with concurrent insulin measurement provides a more informative approach. Evidence suggests that the OGTT without insulin measurement may also indicate an increased likelihood of IR and impaired β-cell function in asymptomatic individuals at risk of type 2 diabetes who are otherwise free of chronic disease [107]. However, the OGTT shows only a moderate correlation with IR as assessed by reference methods, which limits its utility as a standalone diagnostic tool for IR [108]. Indeed, as many as 54–75% of individuals with normal glucose tolerance have been shown to exhibit abnormal insulin response patterns [109,110].

Kraft curves, based on analysis of the insulin response during the OGTT, may help identify hyperinsulinemia and individuals with an increased likelihood of early insulin resistance, even in the presence of normal glucose values. This supports the clinical value of adding insulin measurements to the OGTT. A 2 h OGTT insulin value >30 µU/mL has been proposed as indicative of hyperinsulinemia [54,111]. Another study established a cutoff for IR of 45.68 μU/mL for insulin at 2 h post-OGTT [103]. It is worth noting, however, that measuring both glucose and insulin concurrently during the OGTT may be more informative than measuring either parameter alone. This combined approach enables, among other things, calculation of the Matsuda Index, which provides a good reflection of whole-body insulin sensitivity [112] and improves the identification of individuals with insulin resistance compared with HOMA-IR alone, enabling correct reclassification in as many as 26% of cases [113].

For glucose, well-defined diagnostic criteria exist: a fasting concentration of 100–125 mg/dL indicates prediabetes, and ≥126 mg/dL indicates diabetes, while at 2 h post-OGTT, values of 140–199 mg/dL correspond to impaired glucose tolerance, and ≥200 mg/dL allow a diagnosis of diabetes [114]. In contrast to glucose, no widely accepted norms or cutoff values recommended by scientific societies have yet been established for insulin concentrations measured during the OGTT. Although standardized insulin cut-offs are still lacking, recent evidence indicates that additional information obtained during the OGTT—particularly the 1 h glucose concentration—may also improve the identification of individuals with early dysglycaemia and insulin resistance. Emerging evidence suggests that a 1 h post-load plasma glucose (1 h-PG) threshold of 11.6 mmol/L (209 mg/dL) provides higher diagnostic accuracy for type 2 diabetes than fasting glucose, 2 h glucose, or HbA1c (AUC 0.96–0.98 across validation cohorts) and identifies affected individuals approximately 1.4–1.6 years earlier than the conventional 2 h threshold [115]. An isolated elevation of 1 h-PG (≥8.6 mmol/L [155 mg/dL]) has likewise emerged as an intermediate metabolic phenotype between normal and impaired glucose regulation, in which lifestyle intervention produces particularly pronounced improvements in insulin sensitivity, β-cell function, ectopic fat accumulation, and long-term diabetes risk [116].

Based on Kraft curves, however, fasting, 1 h, and 2 h insulin values of <10 µU/mL, <60 µU/mL, and <30 µU/mL, respectively, may be used as approximate interpretive thresholds. It should be noted, however, that the original Kraft curves were derived from an OGTT using a 100 g glucose load, whereas a 75 g glucose load is now the standard in contemporary practice. A lower glucose load would be expected to elicit a lower insulin response; approximate interpretive values of <10 µU/mL fasting, <50 µU/mL at 1 h, and <30 µU/mL at 2 h post-glucose load have been proposed for clinical interpretation [117], although these thresholds have not been universally standardized.

At the same time, particular caution is warranted when interpreting OGTT results in metabolically healthy individuals following low-carbohydrate or ketogenic diets. Metabolic adaptation to chronically restricted carbohydrate intake can produce seemingly abnormal OGTT results, increasing the risk of misclassification of glucose metabolism status, including diabetes [18,19]. As a result, metabolically healthy individuals may be advised to substantially increase their carbohydrate intake—sometimes to as much as 60% of total energy intake, corresponding to approximately 375 g per day on a 2500 kcal diet [118]—despite having previously functioned well on a low-carbohydrate diet. In such individuals, these recommendations may be unnecessary or poorly tolerated and should be considered in the context of the patient’s dietary pattern, metabolic status, and clinical goals. Additionally, in patients with known or suspected malignancy, the metabolic effects of an OGTT may warrant consideration, as many tumor cells rely heavily on glucose metabolism and may be influenced by insulin-related growth signaling [119]. However, direct clinical evidence demonstrating harm from a single diagnostic OGTT in this population is currently lacking. The rationale for OGTT testing in individuals following low-carbohydrate dietary models is discussed in detail in Section 4.3.

### 6.6. The Triglyceride/High-Density Lipoprotein Cholesterol (TG/HDL-C) Ratio

The TG/HDL-C ratio is another important surrogate marker of IR [120]. An analysis of 32 studies involving nearly 50,000 participants of diverse ethnic backgrounds, including both adults and children, identified the TG/HDL-C ratio as a simple and accessible index of insulin resistance. Proposed cutoff values were 2.53 for women and 2.8 for men, although the authors noted that further research is needed to refine these thresholds—for instance, by sex and ethnic group [121]. Another 2026 publication concluded that TG/HDL-C can be used as an independent marker of IR in settings where fasting insulin measurement and HOMA-IR calculation are not feasible. When insulin measurement and HOMA-IR calculation are available, however, TG/HDL-C may serve as a supportive marker [122]. This index may have additional clinical value, as it can simultaneously serve as a marker of cardiovascular disease (CVD) risk, as documented in multiple studies [123,124]. For example, one study found that a TG/HDL-C ratio ≥2.5 was associated with a higher risk of CVD in patients with type 2 diabetes mellitus (T2DM), retinopathy, and hyperlipidemia, but no prior cardiovascular disease [125]. In clinical practice, some clinicians also use pragmatic interpretive ranges for the TG/HDL-C ratio, considering values <2.0 as favorable, 2.0–3.5 as intermediate, and >3.5 as suggestive of increased cardiometabolic risk [126]. However, these ranges have not been universally validated or endorsed by scientific societies and should therefore be regarded as approximate interpretive thresholds rather than established diagnostic cutoffs for IR.

### 6.7. Continuous Glucose Monitoring (CGM)

Continuous glucose monitoring (CGM) allows real-time monitoring of glucose concentrations throughout the day. These devices consist of a sensor inserted under the skin (secured with an adhesive patch) and a transmitter that relays glucose data to a receiver, such as a smartphone [127,128]. CGM-derived mean glucose values have been shown to correlate with the degree of IR and may help identify glycemic abnormalities earlier than fasting glucose or HbA1c in some individuals [129,130]. Another study reported that glycemic variability, as assessed by CGM, shows a strong positive correlation with IR and may serve as an early indicator of metabolic dysfunction, even in individuals without a diagnosis of diabetes [131]. It should be noted that CGM measures glucose alone; a rapid return to euglycemia following a glucose excursion may reflect either preserved insulin sensitivity or compensatory hyperinsulinemia, a distinction CGM data cannot resolve on their own. Pairing CGM with periodic capillary ketone or insulin measurement may therefore provide a more complete metabolic picture.

### 6.8. TyG-Based Indices (TyG, TyG-BMI, TyG-WHtR)

There are many additional markers that can help detect IR. For example, the triglyceride-glucose (TyG) index, calculated from fasting glucose and triglyceride concentrations, has been shown to be a useful indicator, demonstrating greater predictive performance than HOMA-IR for metabolic syndrome and other IR-related conditions across diverse populations [132]. Authors of a 2026 study found that indices based on the TyG index, when combined with anthropometric parameters, demonstrate even greater diagnostic value. In particular, TyG indices adjusted for body mass index (TyG-BMI) and waist-to-height ratio (TyG-WHtR) showed a strong correlation with IR as assessed by the gold-standard EHC in nondiabetic Japanese adults. The authors explicitly conclude that these indices may serve as practical surrogate markers for IR in situations where use of the EHC is not feasible [133]. Although the TyG index has demonstrated good diagnostic performance as a surrogate marker of IR, reported cut-off values vary considerably across populations and study designs, ranging from 4.49 to 9.45, highlighting the need for population-specific validation [134].

### 6.9. The Alanine Aminotransferase/Aspartate Aminotransferase (ALT/AST) Ratio

The ALT/AST ratio represents an inexpensive, widely available surrogate marker of insulin resistance based on routinely measured liver enzymes. In a study of non-obese Japanese adults, the optimal ALT/AST cutoff for identifying IR (HOMA-IR ≥ 2.5) was ≥0.82 (AUC 0.70), rising to ≥1.02 in overweight individuals [135]. In a large Korean population-based study using KNHANES data (*n* = 11,547), the ALT/AST ratio demonstrated superior predictive performance for HOMA-IR and other IR indices compared with ALT level alone, particularly among women [136]. Consistent associations were reported in a cohort of 459 Japanese women, in which the ALT/AST ratio correlated with HOMA-IR, the Matsuda index, and impaired β-cell function (oral disposition index), with stronger associations observed in middle-aged women compared with young women [137]. Given its low cost and wide availability, the ALT/AST ratio may serve as a practical adjunct to established surrogate indices, particularly in settings where fasting insulin measurement is not feasible; further validation across diverse populations is warranted before specific cutoff values can be recommended for routine clinical use.

### 6.10. The Personalized Insulin Threshold (PIT) Protocol

A recently proposed approach to detecting subclinical hyperinsulinemia relies on serial capillary glucose and ketone (β-hydroxybutyrate, BHB) measurements taken over at least seven consecutive evenings, at least three hours postprandial. According to this framework, consistently suppressed ketone levels (BHB < 0.5 mmol/L) or a glucose-ketone index (GKI, the ratio of glucose to ketones) above 4 are proposed to indicate that circulating insulin has exceeded an individual’s personalized hyperinsulinemia threshold (PIT), resulting in a state termed insulin-compensated euglycemia (ICE)—normoglycemia maintained at the cost of elevated insulin secretion sufficient to suppress ketogenesis [24,138,139]. This is a novel and conceptually interesting approach, consistent with the broader argument of this review that hyperinsulinemia may precede overt glycemic abnormalities by a considerable margin. As a recently introduced method, it would benefit from further research in larger and more diverse populations to establish its diagnostic thresholds and clinical utility alongside established surrogate indices.

No currently available method is suitable for all clinical settings. While the hyperinsulinemic-euglycemic clamp remains the reference standard, its complexity precludes routine clinical use. Consequently, surrogate indices are commonly employed, each offering distinct advantages and limitations with respect to accuracy, accessibility, cost, and clinical applicability. Selection of the most appropriate diagnostic approach should therefore depend on the clinical context, the purpose of assessment, and the resources available.

## 7. Current Clinical Status of Insulin Resistance

Although insulin resistance plays a fundamental role in the pathogenesis of type 2 diabetes, metabolic syndrome, and a range of other chronic and metabolic diseases (discussed in detail in Section 9), it has not yet been formally recognized as a distinct disease entity. In the current World Health Organization (WHO) International Classification of Diseases, 11th Revision (ICD-11) classification, no separate diagnostic code exists for IR [140]. However, ICD-11 includes a category termed “Insulin-resistance syndromes” (code 5A44), which encompasses a group of rare clinical syndromes associated with severe IR [141]. This suggests that IR is currently regarded as a significant metabolic disturbance and a mechanistic contributor to numerous diseases, rather than as an independent clinical diagnosis in its own right.

A similar approach is reflected in the American Diabetes Association (ADA), which identifies IR as one of the most important risk factors for the development of prediabetes and type 2 diabetes, while likewise not treating it as a distinct disease entity. Current ADA guidelines, moreover, do not recommend routine assessment of IR in individuals without a diagnosis of diabetes. In clinical practice, the identification of at-risk individuals relies primarily on the evaluation of glucose metabolism abnormalities and the presence of metabolic risk factors, rather than on direct measurement of the degree of IR [114,142,143]. The current approach to insulin resistance has several important limitations. The absence of a universally accepted clinical definition, standardized diagnostic criteria, and consistent recommendations regarding routine assessment contributes to substantial heterogeneity in both research and clinical practice. As a result, IR is frequently identified only after metabolic complications have already developed, limiting opportunities for early preventive intervention.

At present, IR is therefore regarded primarily as a metabolic disturbance and a significant risk factor for the development of numerous chronic diseases, rather than as a standalone disease entity. This view, however, may be evolving: a growing number of clinicians and researchers worldwide are increasingly describing IR as a distinct disease entity, recognizing it as a distinct pathological process warranting formal diagnosis and treatment. In light of the expanding body of scientific evidence and the potential consequences of its lack of formal recognition, a discussion regarding the revision of the current approach appears warranted. Recognizing IR as a distinct disease entity could facilitate earlier identification of at-risk individuals, enable the implementation of preventive measures at the preclinical stage, and help reduce a range of adverse health and systemic consequences, as discussed further in Section 9.

## 8. Does Insulin Resistance Meet the Criteria for a Distinct Disease Entity?

Despite the well-documented role of insulin resistance in the pathogenesis of numerous chronic diseases, it has not yet been formally recognized as a distinct disease entity. The data summarized below provide grounds for initiating a discussion on revising its current clinical status.

### 8.1. The Evolving Approach to Defining Disease Entities

In recent years, a shift has emerged in the way disease entities are defined. Classification systems are increasingly moving away from frameworks based solely on clinical presentation toward approaches that incorporate shared biological mechanisms, biomarkers, and therapeutic implications. One example of this approach is the concept of immunometabolic depression proposed by Penninx et al. [144], in which the authors argue that delineating biologically coherent phenotypes can provide a foundation for more precise diagnosis and treatment. This paradigm is further reinforced by emerging longitudinal multi-omics approaches. For example, recent proteogenomic analyses suggest that body-fat distribution, IR/HI, and β-cell dysfunction represent biologically distinct drivers of progression from prediabetes to type 2 diabetes, supporting disease classification based on underlying mechanisms rather than clinical phenotype alone [145]. Together, these developments reflect the broader evolution of precision medicine and support a reassessment of the status of disorders with well-characterized pathophysiology. Against this backdrop, it is reasonable to consider whether IR meets the defining characteristics of a distinct disease entity.

### 8.2. Insulin Resistance Evaluated Against the Defining Features of a Disease Entity

Although no formal, universally accepted criteria exist for designating a disorder as a distinct disease entity, clinical medicine generally regards the following as particularly important: identifiable pathophysiology, the capacity for diagnostic detection, a predictable natural course, well-documented clinical consequences, the potential for therapeutic intervention, the presence of impaired tissue or organ function, and clinical utility of the diagnosis [146]. Analysis of the available evidence indicates that pathological IR fulfills each of these criteria, as summarized in Table 2.

The evidence summarized above suggests that in addition to being a risk factor, insulin resistance is a disease state in its own right, one that, if allowed to persist over time, drives pathologically adaptive biology with identifiable biomarkers and far-reaching, though potentially reversible, clinical consequences. This does not imply that the available evidence definitively justifies recognizing IR as a distinct disease entity. However, it does suggest that there are sufficient scientific grounds to initiate a substantive discussion on revising the current approach.

### 8.3. Toward a Practical Clinical Framework for Insulin Resistance

Although the purpose of this review is not to propose a new formal definition of insulin resistance, the available evidence allows several key elements of a future clinical framework to be identified. In our opinion, such a framework should:

1. distinguish physiological from pathological IR;
2. integrate clinical assessment with the most appropriate diagnostic methods, rather than rely on a single test;
3. emphasize early identification before the development of overt metabolic disease;
4. support the future development of standardized diagnostic criteria for clinical practice and research.

These principles are intended to summarize the current evidence rather than to establish a new formal definition of insulin resistance. Further research and expert consensus will be required before a standardized clinical framework can be adopted.

## 9. Consequences of the Underrecognition of Insulin Resistance

Insufficient recognition of insulin resistance in everyday clinical practice—despite its high prevalence and well-documented health consequences—represents an important gap in clinical care, with medical, ethical, and public health implications. Chronically elevated insulin concentrations, closely associated with IR, are linked to an increased risk of numerous chronic diseases. Nevertheless, this condition remains underdiagnosed and is not routinely assessed in many clinical settings. The consequences of this underrecognition are clinically and systemically significant, including rising rates of hospitalization, premature mortality, and escalating healthcare costs. Given the scale of this clinical and public health challenge, recognition of IR and hyperinsulinemia as important and independent risk factors for chronic disease can no longer be postponed. Scientific societies and healthcare institutions will need to consider these disturbances in the design of screening strategies, preventive programs, and public health campaigns aimed at reducing the burden of metabolic and chronic diseases. Insulin resistance has been described as a “silent pandemic”: often undetected in individual clinical encounters, yet increasingly relevant to the burden experienced across healthcare systems. This section outlines the principal consequences arising from its systemic underrecognition.

### 9.1. Delayed Diagnosis and the Absence of Early Metabolic Intervention

Delayed identification of insulin resistance may result in the absence of timely metabolic intervention, leading to significant health consequences, including the development of metabolic disorders and their complications [147,148,149]. This represents one of the most clinically important consequences of underdiagnosis, affecting millions of individuals worldwide. In clinical practice, disorders related to IR are most often identified only once type 2 diabetes—of which IR/HI is a major underlying cause—has already developed [8], or, in a more favorable scenario, at the stage of prediabetes, which may likewise reflect long-standing, previously unrecognized metabolic dysfunction. As noted in Section 6.5, approximately 54–75% of individuals with normal glucose tolerance may exhibit abnormal insulin response patterns that precede overt glycemic abnormalities and could, in principle, be detected at an earlier stage [109,110]. Failure to identify IR may contribute not only to its progression to type 2 diabetes, but also to the development of a broad spectrum of metabolic diseases (discussed in Section 9.2) that could potentially be prevented through sufficiently early diagnosis and the implementation of targeted metabolic intervention. Notably, recent evidence from the Prediabetes Lifestyle Intervention Study showed that remission of prediabetes to normal glucose regulation is achievable independent of weight loss—and even in the context of weight gain—through improvements in insulin sensitivity, β-cell function, and β-cell sensitivity to GLP-1, and that remission is associated with durable protection against incident type 2 diabetes [150]. Long-term post hoc analyses further demonstrated that achieving prediabetes remission was associated with a substantially lower risk of cardiovascular mortality and heart-failure hospitalization, with benefits persisting decades beyond the active intervention period [151].

### 9.2. The Accelerating Cascade of Metabolic Disease

The global burden of metabolic disease has risen substantially over the past two decades, constituting one of the defining public health challenges of the present era [152,153]. Insulin resistance emerges as the common denominator and a key pathogenic factor underlying a substantial proportion of these conditions, a relationship well established in the literature [154,155]. One study estimated the proportion of American adults exhibiting optimal cardiometabolic health, finding that only 12.2% of the population met these criteria [156]—a finding that may point to significant limitations in current strategies for the prevention and early intervention of metabolic disorders.

#### 9.2.1. Type 2 Diabetes

Type 2 diabetes rarely develops in the absence of insulin resistance, which often precedes the clinical onset of the disease by 10 to 15 years [8]. This implies that early detection of IR, coupled with the timely initiation of metabolic therapy, could substantially reduce the future incidence of T2DM worldwide—and, in turn, the millions of complications that result from it. Current prevalence statistics for T2DM are nonetheless alarming, and projections for the coming years are even more concerning. An estimated 589 million adults aged 20–79 years are currently living with diabetes worldwide, corresponding to roughly one in nine individuals within this age group. Projections indicate that this figure may rise to approximately 853 million by 2050. Moreover, the disease imposes an enormous economic burden: global healthcare expenditure related to diabetes is estimated to have exceeded US $1 trillion in 2024, representing an increase of approximately 338% over the past 17 years [157].

#### 9.2.2. Metabolic Dysfunction-Associated Steatotic Liver Disease (MASLD)

Insulin resistance is central to the development of metabolic dysfunction-associated steatotic liver disease (MASLD), whose global prevalence is estimated at approximately one in three individuals [158,159]. Although the prevalence of IR in MASLD may be underestimated because of the lack of standardized diagnostic criteria, IR is present in the majority of patients, affecting approximately 80% of cases, including individuals with normal BMI, and exceeding 90% in more advanced disease stages [160,161,162,163]. Accordingly, up to 80.6% of patients with type 2 diabetes have concomitant MASLD [164]. Despite this high prevalence, one study reported that 96% of adults with MASLD in the United States were unaware of their condition [165].

Insulin resistance disrupts glucose and lipid metabolism, promoting compensatory hyperinsulinemia, hepatic de novo lipogenesis, increased free fatty acid influx, inflammation, and ultimately hepatic triglyceride accumulation [159,166,167,168]. Recent studies further highlight the role of hepatic IR in disease progression and suggest that excess dietary fructose may accelerate hepatic fat accumulation, whereas essential phospholipids may provide benefit as an adjunct to lifestyle intervention [169,170]. The absence of widespread screening for IR limits opportunities for early identification and prevention of MASLD progression.

#### 9.2.3. Metabolic Syndrome (MetS)

IR is a key pathophysiological component of metabolic syndrome (MetS), which is characterized by the coexistence of abdominal obesity, dyslipidemia, hypertension, and insulin resistance, with diagnosis based on the presence of at least three of these factors [171,172]. MetS has become a major global health challenge: approximately 39.8% of adults in the United States meet the diagnostic criteria, rising to 56.4% among individuals aged ≥60 years [173,174]. Globally, an estimated 1.54 billion adults had MetS in 2023, with prevalence more than doubling among women and nearly tripling among men since 2000 [175]. Early identification of IR/HI is therefore of particular importance, as longitudinal changes in IR/HI indices have been shown to predict the future development of MetS and may facilitate earlier identification of high-risk individuals [149].

#### 9.2.4. Hypertension (HTN)

Hypertension (HTN) is one of the leading causes of premature mortality worldwide and affects more than 1 billion people globally [176,177,178]. Among individuals aged 30–79 years, the number of people with hypertension nearly doubled between 1990 and 2019, rising from 331 million to 626 million women and from 317 million to 652 million men—an increase driven primarily by population growth and aging, as global age-standardized prevalence remained relatively stable over this period [179]. Recent studies increasingly implicate IR as an important contributor to HTN. A 2026 study found that each one-unit increase in the TyG index, TG/HDL-C ratio, METS-IR, and CHG index was associated with a 23.4%, 8.1%, 52.5%, and 9.9% higher risk of HTN, respectively [180]. IR promotes hypertension through endothelial dysfunction, reduced nitric oxide bioavailability, increased vascular resistance, volume overload, and activation of the sympathetic nervous system and the renin–angiotensin–aldosterone system [51,181,182,183,184,185,186,187,188]. Consequently, failure to identify IR early may delay recognition of the metabolic abnormalities contributing to HTN.

#### 9.2.5. Cardiovascular Disease (CVD)

Although hypertension is an important component of cardiovascular disease (CVD), the impact of IR extends far beyond blood pressure regulation. Increasing evidence indicates that IR and the associated metabolic dysfunction contribute to the development of a wide range of cardiovascular diseases and have long represented an underrecognized component of CVD [189,190,191]. IR has also been associated with subclinical atherosclerosis in individuals with normal glycemia, suggesting a role in the early stages of disease development [192]. Proposed mechanisms include chronic inflammation, lipotoxicity, dyslipidemia, oxidative stress, endothelial dysfunction, and hypertension, all of which promote atherosclerosis and cardiovascular damage [193]. In addition, individuals with the greatest long-term variability in insulin concentrations have been shown to have a 65% higher risk of CVD and a 97% higher risk of all-cause mortality, further emphasizing the importance of early identification and management of insulin dysregulation [194].

#### 9.2.6. Cellular Senescence and Cancer

Cancer is the second leading cause of death worldwide, accounting for approximately one in six deaths globally and nearly 20 million new cases annually [195,196]. A growing body of evidence links IR to multiple cancers. A 2026 study reported associations between IR and 12 cancer types, with particularly strong relationships observed for endometrial, renal, esophageal, pancreatic, colorectal, and breast cancer [197]. Another study found that IR, combined with chronic inflammation, increased cancer risk by as much as 71% [198]. Proposed mechanisms include activation of the insulin/IGF axis, chronic inflammation, metabolic reprogramming, and mitochondrial dysfunction, all of which may promote tumor initiation and progression [199].

**Figure 5:**
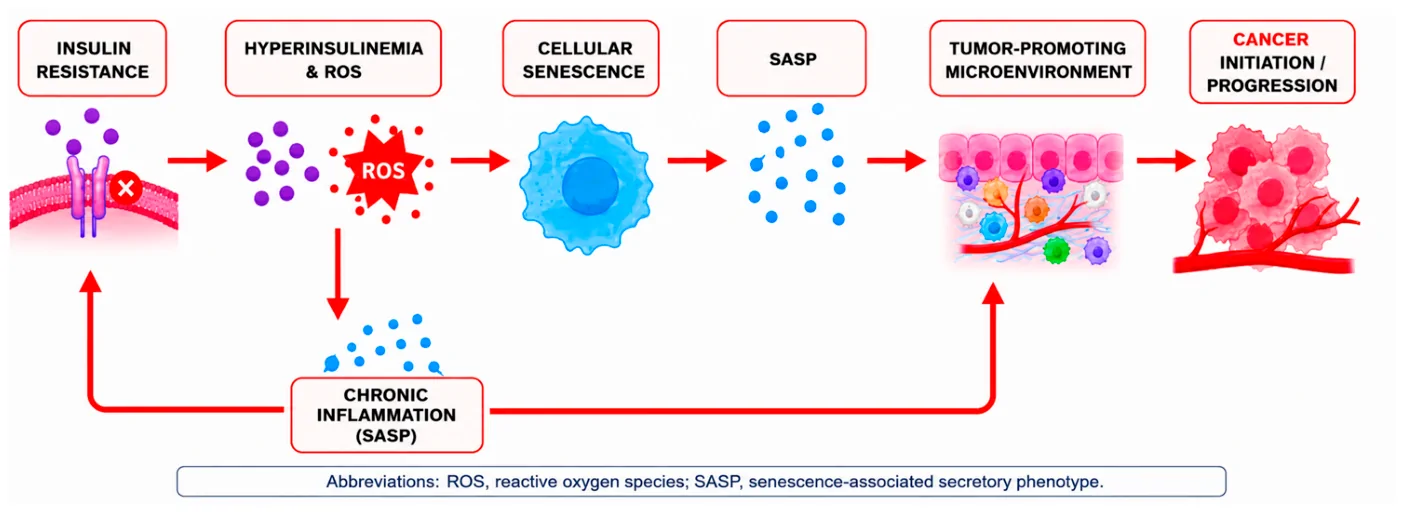
Mechanistic interplay between insulin resistance, cellular senescence, chronic inflammation (SASP), and cancer development.

Emerging evidence further suggests that cellular senescence may represent an additional mechanistic link between IR and cancer. Chronic hyperinsulinemia, oxidative stress, and metabolic dysfunction promote the accumulation of senescent cells and the development of a pro-inflammatory senescence-associated secretory phenotype (SASP), which may contribute to chronic inflammation, IR, and a pro-tumorigenic microenvironment [200,201,202,203,204,205,206]. Consequently, failure to identify IR early may limit opportunities for timely metabolic intervention and, potentially, prevention of a subset of cancers. It should also be noted that glucose-based diagnostic testing, such as the OGTT, warrants particular caution in patients with known or suspected malignancy—hyperglycemia is a well-documented trigger of seizures [207], a mechanism of particular concern in patients with brain tumors, who are already predisposed to seizure activity—making fasting-based, non-glucose-load assessments preferable in this population. The proposed interplay between IR, cellular senescence, chronic inflammation, and cancer is summarized in Figure 5.

#### 9.2.7. Neuropsychiatric Disorders

The emerging field of metabolic psychiatry highlights a close association between systemic IR and psychiatric disorders, including depression, bipolar disorder, and schizophrenia [208,209]. Because the brain has exceptionally high glucose demands, impaired insulin signaling may adversely affect neuroplasticity, neurotransmitter regulation, and cognitive function. Growing evidence further suggests that a subset of treatment-resistant psychiatric disorders may have an underlying metabolic basis [208,209,210,211,212,213,214]. Greater recognition of this relationship may improve the identification of patients who could benefit from metabolic interventions, representing another important clinical implication of early IR detection.

### 9.3. Increased Mortality

The increased mortality resulting from delayed or insufficient recognition of IR at an early stage is directly linked to Section 9.2. While the escalating cascade of metabolic disease substantially affects the quality of life of millions of people worldwide, it may ultimately contribute to premature death—deaths that, in many cases, could potentially have been avoided. In 2024, for instance, diabetes was a major cause of premature mortality, accounting for approximately 3.4 million deaths worldwide—one death every 9 s [157]. Cardiovascular disease currently represents the leading cause of death globally, estimated to have accounted for nearly 20 million deaths in 2022, or approximately 32% of all deaths worldwide. Of these deaths, as many as 85% were attributable to myocardial infarction and stroke. Notably, a substantial proportion of these events are preventable through lifestyle modification and risk factor control [215]. Hypertension alone is among the leading contributors to premature mortality worldwide [177,216]. Cancer also accounts for 1 in 6 deaths globally, placing it—alongside CVD—among the leading causes of death worldwide [195], with an estimated 630,000 deaths projected in the United States alone in 2026 [217]. MASLD, which affects as many as 1 in 3 individuals, substantially increases mortality risk by as much as 35–92% compared with individuals without the disease [218,219], with elevated risk observed even among lean individuals with MASLD [220]. Similarly, metabolic syndrome increases all-cause mortality risk by 24%, heart disease mortality risk by 44%, and diabetes-related mortality risk by as much as fivefold [221]. Even assuming that only a fraction of these deaths could be averted through sufficiently early identification of IR/HI, the scale of potential health benefit remains substantial, pointing to the possibility of preventing—or at least delaying—millions of premature deaths worldwide.

### 9.4. The Absence of Systemic Metabolic Intervention

Section 9.1 discussed the problem of delayed metabolic intervention stemming from the underdiagnosis of insulin resistance. It should be emphasized, however, that the problem extends beyond delayed diagnosis: even when IR is identified, standardized, systemic management strategies remain lacking, with current clinical attention focused primarily on its late-stage manifestations, such as type 2 diabetes [222]. The existence of a dedicated ICD code enables the formal recognition of a clinical diagnosis, providing the foundation for the implementation of standardized diagnostic and therapeutic pathways [140,223]. In clinical practice, medical decision-making is largely organized around disease entities captured within the ICD classification [224]. Accordingly, the absence of a dedicated code for IR limits its formal recognition as a condition warranting intervention. As a consequence, systemic reporting and analysis of therapeutic interventions within the framework of the International Classification of Health Interventions (ICHI) are similarly hindered, further limiting the ability to evaluate and standardize clinical management in this area [223]. The lack of a formal classification for IR also substantially complicates its systematic monitoring and long-term patient management. The introduction of a dedicated ICD code for IR could therefore help redirect healthcare system efforts toward earlier detection of this disorder and the implementation of targeted metabolic interventions aimed at preventing progression or promoting remission. The absence of a dedicated code, however, should not preclude clinicians from actively identifying and managing IR—it remains a metabolic disease with real, measurable consequences for patients, warranting clinical attention regardless of its current classificatory status. Even in the absence of a dedicated ICD code, clinicians are encouraged to identify, document, and actively manage IR and hyperinsulinemia, working toward metabolic remission with a progressive reduction in medication burden over time.

### 9.5. Lack of Reimbursement for Diagnostic Testing

The absence of formal recognition of IR as a disease entity may limit the development of standardized diagnostic pathways for its identification and, consequently, reimbursement mechanisms for such testing. Coding systems, including diagnostic codes (ICDs) and procedural codes, such as the Current Procedural Terminology/Healthcare Common Procedure Coding System (CPT/HCPCS), form the foundation for billing health services and processing payer claims [225]. Within reimbursement systems based on the Diagnosis-Related Groups (DRG) model, payment amounts are often determined by the assigned diagnostic group, which is in turn dictated by ICDs, making them a central element of healthcare billing [226].

The absence of funding for diagnostic services may result in their restricted use or omission for economic reasons. This, in turn, delays the identification of IR, which, if detected sufficiently early under conditions of reimbursed diagnostic testing, could be addressed through interventions aimed at preventing progression to further metabolic disturbances and chronic disease.

### 9.6. Limited Epidemiological Reporting

**Figure 6:**
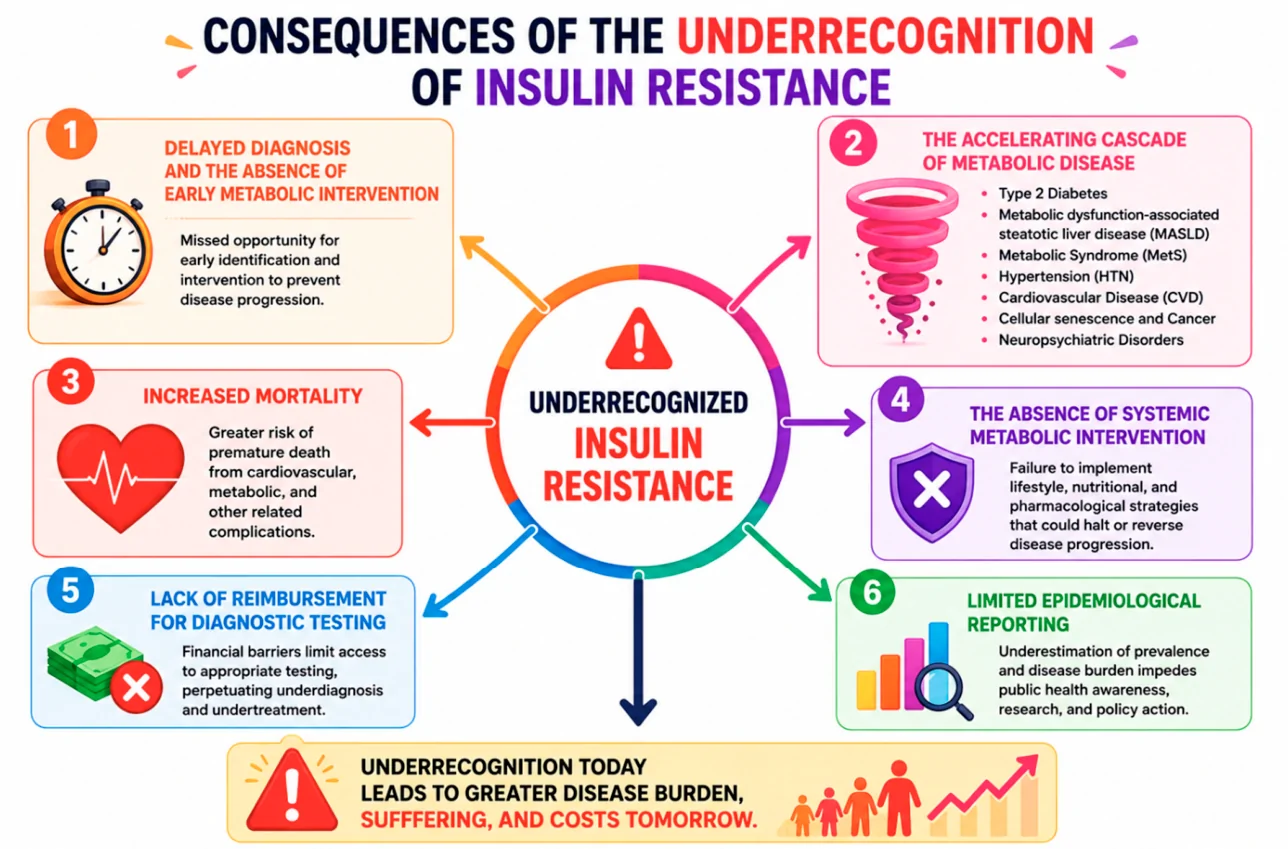
Consequences of the Underrecognition of Insulin Resistance.

The ICD serves as a fundamental tool in epidemiology and public health surveillance [227]. Accurate coding supports the monitoring of incidence and prevalence, the tracking of healthcare outcomes, and the evaluation of public health interventions. As such, it underpins the collection, processing, and reporting of data that are essential for informed health policy and decision-making. It also enables the pooling and comparison of data across different systems and regions, facilitating the analysis of epidemiological trends, the identification of disease clusters, and the assessment of health disparities within populations [140,227]. As noted in Section 1, however, considerable discrepancies currently exist in reported IR prevalence, ranging from 15.5% to as high as 61.2%, with an averaged global estimate of 26.53% [1,2,147]. Despite these already concerning estimates, the true prevalence of IR is likely underestimated, reflecting the absence of widespread diagnosis and standardized diagnostic criteria [8,228]. Limited epidemiological reporting of IR may therefore not only contribute to its underestimation but also delay preventive action, distort population-level health risk assessment, and impede sound clinical and systemic decision-making. Consequences of the underrecognition of IR are illustrated in Figure 6.

## 10. Treatment of Insulin Resistance

The goal of treating IR is to reduce chronic hyperinsulinemia, improve insulin sensitivity, and prevent metabolic and organ-related complications. Current evidence indicates that dietary modification—particularly carbohydrate restriction, often combined with reduced energy intake and regular physical activity—represents a cornerstone of IR management [191,229,230,231,232,233]. A randomized controlled trial demonstrated that a daily energy deficit of approximately 400 kcal significantly improved glucose tolerance, insulin sensitivity, body composition, and liver fat content over six months. Additional reduction in red meat intake or increased fiber consumption did not provide further metabolic benefits, suggesting that caloric restriction itself may be the principal driver of these improvements [234]. Intermittent fasting may also be beneficial [127,235]. Physical activity should be individualized according to patient preferences, as enjoyment and intrinsic motivation improve long-term adherence [236,237,238]. Beyond increasing energy expenditure, exercise improves insulin sensitivity through favorable changes in skeletal muscle metabolism, including enhanced glucose uptake, fatty acid oxidation, mitochondrial function, and myokine secretion [239,240]. Holten et al. further demonstrated that only six weeks of resistance training increased skeletal muscle GLUT4 protein content by approximately 40% in patients with type 2 diabetes, accompanied by increased insulin-stimulated glucose uptake and enhanced insulin signaling, independent of substantial muscle hypertrophy [241]. Psychological factors should also be considered an integral component of IR management. Chronic stress contributes to IR through persistent activation of the hypothalamic–pituitary–adrenal axis and elevated cortisol concentrations, increasing the risk of progression to T2DM [33,242,243,244,245,246,247]. Accordingly, stress-reduction strategies—including mindfulness, cognitive behavioral therapy, and regular exposure to natural environments—may complement dietary and lifestyle interventions [248,249,250,251,252].

Collaboration with a psychologist or psychotherapist may be particularly beneficial for patients with disordered eating, compulsive overeating, or food addiction, all of which can compromise long-term treatment success. Food addiction has been increasingly recognized as sharing neurobiological features with substance addiction, with highly processed foods activating dopaminergic reward pathways and promoting loss of control over eating behavior [253,254,255]. A large meta-analysis found that food addiction was associated with a twofold higher risk of type 2 diabetes, independent of body weight [256]. Combining low-carbohydrate dietary interventions with psychological and group-based support has also been shown to improve metabolic outcomes and reduce symptoms of food addiction [257].

Although no medications are currently approved specifically for the treatment of IR, several drugs used in type 2 diabetes—including metformin, sodium-glucose cotransporter-2 (SGLT-2) inhibitors, and glucagon-like peptide-1 receptor agonists (GLP-1RAs)—may improve insulin sensitivity and reduce compensatory hyperinsulinemia. Certain supplements, particularly berberine, L-arginine, and magnesium, may also provide adjunctive benefits, although further high-quality studies are required [191]. Magnesium status has been inversely associated with insulin resistance, particularly in individuals with deficiency or impaired glucose homeostasis, through proposed effects on insulin signaling and GLUT4-mediated glucose uptake [258,259]. Other dietary bioactive compounds, including omega-3 polyunsaturated fatty acids (PUFAs) and anti-inflammatory polyphenols, which may improve insulin sensitivity through mechanisms including suppression of NF-κB, JNK, and IKKβ signaling [260], as well as probiotic and prebiotic supplementation, which has been associated with reductions in HOMA-IR in systematic reviews and meta-analyses [261], may provide additional adjunctive benefit, although current evidence remains preliminary.

## 11. Limitations

This review has several limitations. As a narrative review, it does not follow the methodology of a systematic review and is therefore subject to the inherent limitations of narrative evidence synthesis. In addition, the topics discussed are supported by evidence of varying strength, ranging from well-established findings to emerging concepts that require further investigation. Accordingly, the purpose of this review is not to propose a new formal definition of insulin resistance or to definitively establish its status as a distinct disease entity, but rather to critically evaluate the available evidence and identify areas requiring further research and expert consensus. It should also be acknowledged that the evidence discussed throughout this review is open to alternative interpretation, and that considerable disagreement currently exists among experts regarding whether the available evidence is sufficient to support formal disease-entity status for insulin resistance. The perspective offered here reflects the authors’ synthesis of the current literature and is intended to contribute to this ongoing discussion rather than to resolve it.

## 12. Summary

Taken together, the evidence presented in this review supports several key conclusions:

1. Insulin resistance (IR) has reached the scale of a major global public health problem. Given its high prevalence and broad clinical impact, earlier recognition and management of IR should be considered a public health priority.
2. IR is a heterogeneous condition and is not invariably pathological. Physiological insulin resistance occurs during puberty, pregnancy, and—reflecting a distinct, hyperinsulinemia-independent mechanism—as an adaptive reduction in glucose disposal under certain dietary conditions (e.g., carbohydrate restriction), emphasizing the importance of clinical context when interpreting diagnostic findings.
3. IR develops through multiple interacting mechanisms. Current evidence supports roles for hyperinsulinemia, inflammation, stress, sleep disturbances, and dietary factors, although the causal relationships among these mechanisms remain an area of ongoing investigation.
4. The diagnosis of IR remains challenging. While the euglycemic-hyperinsulinemic clamp remains the reference standard, its limited clinical applicability necessitates the use of surrogate markers. Greater standardization of diagnostic approaches is needed to facilitate earlier and more consistent identification of IR.
5. Failure to identify IR at an early stage contributes to delayed metabolic intervention and increases the risk of progression to multiple metabolic and cardiometabolic disorders, including type 2 diabetes, MASLD, metabolic syndrome, hypertension, and cardiovascular disease.
6. Lifestyle interventions remain the cornerstone of IR management, with dietary modification, regular physical activity, and weight reduction forming the basis of current therapeutic strategies.
7. Current evidence supports further consideration of insulin resistance as a distinct clinical entity. Formal recognition could improve diagnostic standardization, facilitate screening, promote earlier metabolic intervention, and strengthen epidemiological surveillance.
8. Regardless of its future disease classification, earlier identification and management of IR may represent one of the most effective strategies for reducing the growing global burden of metabolic disease.

## 13. Conclusions

The evidence presented in this review indicates that insulin resistance exhibits many of the defining features of a distinct clinical entity, including a well-characterized pathophysiology, an emerging capacity for diagnostic identification, a natural course that remains incompletely characterized, and numerous well-documented clinical consequences. However, important challenges remain, particularly regarding the lack of standardized diagnostic criteria and the absence of broad clinical consensus concerning its formal classification. Current evidence supports the need for a clearer clinical and diagnostic framework for insulin resistance to facilitate its earlier recognition and more consistent management in clinical practice. Greater emphasis on the early identification and treatment of insulin resistance could contribute to more effective prevention of type 2 diabetes, metabolic dysfunction-associated steatotic liver disease, cardiovascular disease, and other metabolic complications. Formal recognition of insulin resistance as a distinct clinical entity may support the standardization of diagnostic and therapeutic approaches, facilitate the implementation of screening strategies, and strengthen epidemiological surveillance. Given the steadily rising prevalence of metabolic disorders, earlier identification and management of insulin resistance may represent an important component of strategies aimed at reducing the burden and mortality associated with metabolic disease.

## Figures and Tables

**Table 1:** Key Methods for the Assessment of Insulin Resistance.

Method	Advantages	Disadvantages	Clinical Application
EHC Euglycemic Hyperinsulinemic Clamp	Gold standard—highest accuracy Direct measurement of tissue glucose uptake M-value as a precise indicator of IR	Very costly and time-consuming Requires specialized infrastructure Invasive—requires insulin and glucose infusion Not available in routine practice	Used exclusively for research purposes Reference standard for other indices Cutoff: M-value < 5 mg/kg/min
HOMA-IR Homeostatic Model Assessment of IR	Simple formula: (glucose × insulin)/22.5 Inexpensive and widely accessible Commonly used in research	No single, universally accepted global cutoff Cutoff values vary by population and laboratory method Reported cutoff range: 1.7–3.875, depending on the study Does not capture postprandial response	Screening for IR in clinical practice Assessment of type 2 diabetes risk Typical threshold: 2.5 in adults; 3.6 in children
HOMA-2 Homeostasis Model Assessment 2	Accounts for nonlinear relationships Assesses IR, β-cell function, and insulin sensitivity Greater mathematical accuracy than HOMA1	No widely accepted cutoff values Conflicting evidence versus HOMA1 for predicting T2DM Less commonly used in clinical practice	Scientific and clinical research Assessment of β-cell function (HOMA2-%B) Assessment of insulin sensitivity (HOMA2-%S)
Fasting Insulin Fasting Insulin	Least expensive and most accessible marker of IR Strong correlation with EHC Recognized as a key marker by the DGAC 2025–2030 [9] Effective across diverse populations, including individuals with obesity	No single, universal diagnostic threshold Results vary by sex, age, degree of obesity, and laboratory method Laboratory reference ranges ≠ metabolically optimal range Does not reflect postprandial response	Screening and risk stratification for IR Desirable range: <8 µU/mL (men), <10 µU/mL (women) Values > 15 mU/L—warning sign of metabolic dysfunction Used in conjunction with HOMA-IR and QUICKI
OGTT + insulin Oral Glucose Tolerance Test	Assessment of dynamic glucose response Enables calculation of the Matsuda Index Detects hyperinsulinemia despite normal glycemia Kraft curves—early identification of IR	No widely accepted reference norms for insulin during OGTT Moderate correlation with reference methods False results possible in individuals following low-carbohydrate diets Time-consuming (2–3 h)	Diagnosis of prediabetes and diabetes Approximate insulin thresholds (75 g load): <10 µU/mL (0 h), <50 µU/mL (1 h), <30 µU/mL (2 h) Matsuda Index provides a 26% net reclassification improvement versus HOMA-IR alone
TG/HDL-C Triglyceride-to-HDL ratio	Simple and inexpensive—derived from a routine lipid panel Serves simultaneously as a marker of IR and CVD risk Validated in a meta-analysis of 32 studies (~50,000 participants)	Lower specificity than HOMA-IR Cutoff values vary by sex and ethnicity Recommended as a supportive marker rather than a primary diagnostic tool	When insulin measurement is not feasible Cutoffs: ≥2.53 (women), ≥2.8 (men) Assessment of CVD risk in patients with T2DM
CGM Continuous Glucose Monitoring	24 h glucose monitoring Early identification of glycemic abnormalities Detects IR earlier than fasting glucose or HbA1c Assesses glycemic variability as a marker of IR	Costly compared with blood-based tests Measures glucose, not insulin, directly Limited standardization in the interpretation of results for IR assessment	Early screening for metabolic dysfunction Monitoring of individuals without a diagnosed diabetes Assessment of glycemic variability as an indicator of IR
TyG/TyG-BMI/TyG-WHtR Triglyceride-Glucose Index	TyG index: better predictive performance for metabolic syndrome than HOMA-IR TyG-BMI and TyG-WHtR: strong correlation with EHC Easy to calculate from routine laboratory tests	Less extensively studied than HOMA-IR Requires anthropometric data (BMI, waist-to-height ratio) No widely established cutoff values	Practical surrogate marker for IR when EHC is not feasible Assessment of metabolic syndrome risk TyG-WHtR particularly useful in nondiabetic adults
ALT/AST Alanine Aminotransferase/Aspartate Aminotransferase Ratio	Inexpensive and widely available—based on routinely measured liver enzymes	No single recommended cutoff—reported thresholds vary by population and adiposity status (0.82–1.02); requires further validation across diverse populations	Adjunct to established surrogate indices, particularly when fasting insulin measurement is not feasible; correlates with HOMA-IR, Matsuda index, and β-cell function

**Table 2:** Assessment of Insulin Resistance Against the Defining Features of a Distinct Disease Entity.

Defining Feature of a Disease Entity	Insulin Resistance	Discussed in This Article
Identifiable pathophysiology	✔	Section 5
Capacity for diagnostic detection	✔ (with caveats)	Section 6
Characteristic natural course	✔ (with caveats)	Section 5 and Section 8
Clinical and prognostic consequences	✔	Section 9
Potential for treatment or prevention	✔	Section 10
Impaired tissue and organ function	✔	Section 5
Clinical utility of the diagnosis	✔ (with caveats)	Section 6, Section 9 and Section 10

✔ reflects the authors’ assessment that, based on the evidence reviewed, insulin resistance is broadly consistent with this defining feature of a disease entity, as discussed in the corresponding section(s); “(with caveats)” indicates that this assessment is subject to important limitations or areas of ongoing scientific uncertainty, as detailed in the referenced section(s). This table reflects a synthesis of current evidence rather than a definitive or universally accepted classification.

## Data Availability

No new data were created or analyzed in this study. Data sharing is not applicable to this article.
